# Hypertrophy of human embryonic stem cell–derived cardiomyocytes supported by positive feedback between Ca^2+^ and diacylglycerol signals

**DOI:** 10.1007/s00424-019-02293-0

**Published:** 2019-06-28

**Authors:** Christine Deisl, Michael Fine, Orson W. Moe, Donald W. Hilgemann

**Affiliations:** Departments of Physiology and Internal Medicine, Charles and Jane Pak Center of Mineral Metabolism and Clinical Research, University of Texas Southwestern Medical Center, 5323 Harry Hines Blvd, Dallas, TX 75235 USA

**Keywords:** Diacylglycerol lipase, DO34, Cardiac hypertrophy, Cardiac glycosides, Ouabain, Sodium potassium pump, Patch clamp, Angiotensin-2, Endothelin-1

## Abstract

Human embryonic stem cell–derived cardiomyocytes develop pronounced hypertrophy in response to angiotensin-2, endothelin-1, and a selected mix of three fatty acids. All three of these responses are accompanied by increases in both basal cytoplasmic Ca^2+^ and diacylglycerol, quantified with the Ca^2+^ sensor Fluo-4 and a FRET-based diacylglycerol sensor expressed in these cardiomyocytes. The heart glycoside, ouabain (30 nM), and a recently developed inhibitor of diacylglycerol lipases, DO34 (1 μM), cause similar hypertrophy responses, and both responses are accompanied by equivalent increases of basal Ca^2+^ and diacylglycerol. These results together suggest that basal Ca^2+^ and diacylglycerol form a positive feedback signaling loop that promotes execution of cardiac growth programs in these human myocytes. Given that basal Ca^2+^ in myocytes depends strongly on the Na^+^ gradient, we also tested whether nanomolar ouabain concentrations might stimulate Na^+^/K^+^ pumps, as described by others, and thereby prevent hypertrophy. However, stimulatory effects of nanomolar ouabain (1.5 nM) were not verified on Na^+^/K^+^ pump currents in stem cell–derived myocytes, nor did nanomolar ouabain block hypertrophy induced by endothelin-1. Thus, low-dose ouabain is not a “protective” intervention under the conditions of these experiments in this human myocyte model. To summarize, the major aim of this study has been to characterize the progression of hypertrophy in human embryonic stem cell–derived cardiac myocytes in dependence on diacylglycerol and Na^+^ gradient changes, developing a case that positive feedback coupling between these mechanisms plays an important role in the initiation of hypertrophy programs.

## Introduction

**Fig. 1:**
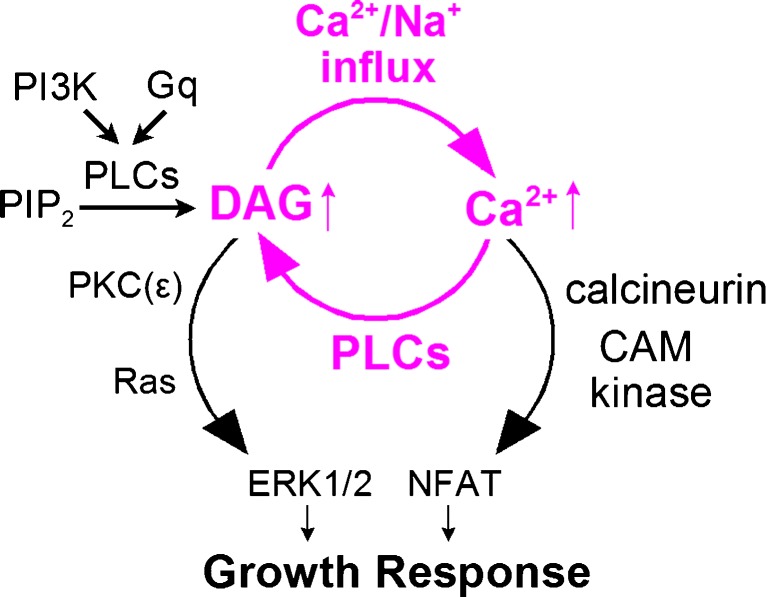
Positive feedback between DAG and Ca^2+^ signaling in the initiation of cardiac hypertrophy. DAG acts to increase Ca^2+^ influx via voltage-gated Ca^2+^ channels as well as both Ca^2+^ and Na^+^ influx via TRPC channels. A rise in basal Ca^2+^ increases PLC activities that are initially enabled by growth factor (PI3K) and GPCR (Gq) signaling. Growth responses are activated coordinately by ERK1/2 and NFAT signaling coupled to PKC/Ras activation and Ca^2+^-activated calcineurin/CAM kinase actions, respectively

Pathological cardiac myocyte hypertrophy [20] is a common step in the progression of heart disease to cardiac failure, a major cause of death worldwide [66]. Signaling programs that promote hypertrophy are activated by multiple mechanical, metabolic, and humoral perturbations. Initiating factors include hypertension, both pulmonary and systemic, myocardial infarction, coronary artery disease, mutations of sarcomeric proteins, diabetic and metabolic cardiomyopathy, viral and bacterial myocarditis, valve insufficiency, congenital heart defects, and chronic abuse of illicit drugs such as cocaine and amphetamines [75]. Hypertrophic growth of myocytes is promoted by the engagement of overlapping, self-amplifying signaling mechanisms. Signaling pathways leading to the activation of nuclear factor of activated T-cell (NFAT) transcription factors and extracellular signal–regulated kinases (ERKs) become intertwined in the activation of hypertrophy programs [69], and a simplified signaling diagram is presented in Fig. 1. In brief, an increase in DAG will increase basal Ca^2+^ in cardiac myocytes by multiple mechanisms, and an increase in basal Ca^2+^ can increase DAG by multiple mechanisms. Details of this coupling and the major signaling pathways leading to cardiac myocyte growth are summarized subsequently.

In the early stages, both Ca^2+^- and DAG-dependent mechanisms are definitively engaged. Local and global Ca^2+^ signals are implicated to promote growth programs via the NFAT transcription factors, whose phosphorylation state and presence in the cytoplasm are under control of Ca^2+^/calmodulin (CAM)-dependent protein kinases and the Ca^2+^-dependent phosphatase, calcineurin [28]. Accumulations of DAG, on the other hand, promote the execution of growth programs via the activation of multiple protein kinase C (PKC) isoforms [45], especially PKCε [32, 72], leading to the activation of the proto-oncogene, Ras, and subsequently ERKs, which phosphorylate additional transcriptional effectors and modify NFAT signaling [69]. Notably, DAG-binding proteins known as Ras guanyl–releasing proteins (RasGRPs), which activate Ras after directly binding DAG at C1 domains, do not seem to be present in cardiac myocytes [74].

The molecular and cellular coupling of DAG- and Ca^2+^-dependent signals represents a potentially very powerful positive feedback loop in the initiation of hypertrophy responses. Most DAG that activates PKCs is generated by phospholipase C (PLC) activities under the control of G protein–coupled receptors (GPCRs) and growth factor receptors [49]. The activation of all PLCs described to date in mammalian cells [49] becomes amplified by the generation of Ca^2+^ signals during which PLC activity is promoted by the direct binding of Ca^2+^ to C2 domains [52] of PLCs [77]. Local Ca^2+^ signals generated by IP_3_-dependent Ca^2+^ release at the nuclear envelope are suggested to become importantly involved [2, 50, 57]. That DAG signaling plays a key role in the initiation of hypertrophy is demonstrated, for example, by findings that the presence or absence of DAG kinase activities, which terminate DAG signals, decisively suppresses and supports, respectively, the execution of hypertrophy programs [3, 54, 55].

That DAG signaling promotes Ca^2+^ signaling, as part of its role in hypertrophy signaling in myocytes, is also unambiguously established. First, L-type Ca_V_ Ca^2+^ channel activity has been shown to be specifically enhanced by DAG generated within the cytoplasm, as opposed to exogenous DAG applied to the outside of cells [26]. Second, and possibly more importantly, nonselective cation channels of the TRPC type are directly activated by DAG, thereby promoting Ca^2+^ and Na^+^ influx that is demonstrated to be essential for the initiation of hypertrophy programs [17, 51, 58]. This role may involve Ca^2+^ signals generated directly by the influx of Ca^2+^ through TRPC channels, and it may involve the indirect enhancement of global myocyte Ca^2+^ signaling via cytoplasmic Na^+^ loading with concomitant Ca^2+^ loading via Na^+^/Ca^2+^ exchange [29].

It would exceed drastically the scope of this article to consider mechanisms of cytoplasmic Na^+^ and heart glycoside signaling beyond Na^+^ gradient coupling to Ca^2+^ via Na^+^/Ca^2+^ exchange. It must suffice here to point out that a great deal of evidence in fact supports the idea that Na^+^/K^+^ pumps can act as signaling molecules with interactions to other proteins that are highly dependent on heart glycosides [4, 16, 41, 53, 60, 71, 76, 79–81]. In spite of this mushrooming literature, it appears essential to us to initiate this study with the simplest possible hypothesis suggested by the principle of Occams’s razor: Heart glycosides definitively increase cardiac contractility by a mechanism that requires the NCX1 Na^+^/Ca^2+^ exchanger [64] and therefore a change of the Na^+^ gradient. Interventions that inhibit constitutive Na influx, such as NHE1 Na^+^/H^+^ exchange knockdown [36], have opposite effects. Na^+^ influx in response to stretch of cardiac myocytes is documented to increase and likely mediate the initiation of hypertrophy programs [35]. Given evidence already outlined that DAG signals enhance Ca^2+^ and Na^+^ influx in cardiac myocytes; the simple hypothesis clearly emerges that hormonal activators of hypertrophy programs, such as endothelin-1 (ET-1), angiotensin-2 (AT-2), and catecholamines, promote joint Ca^2+^ and DAG signaling that becomes linked in a positive feedback loop that supports the initiation of cardiac hypertrophy programs.

The cardiac myocyte models commonly employed in hypertrophy studies, such as cultured adult and neonatal rodent myocytes, have distinct limitations. These include questions about their relevance to human myocyte function, the control of key signaling variables, e.g., spontaneous activity, and finally, their limited viability in culture which often limits chronic experiments. Accordingly, we have chosen to employ human embryonic stem cell (hESC)–derived cardiomyocytes to address the working hypothesis outlined above. hESC-derived myocytes are cost-effective [38, 46] and presently appear useful for studies of cardiac development, function, and pathophysiology [12]. All major features of Ca^2+^ handling in hESC-derived myocyte cultures have been shown to be qualitatively comparable to adult myocytes [31, 39, 63, 70]. However, the excitation–contraction cycle in general occurs on a longer time scale, and this has been interpreted to be an “immature functional phenotype” [37]. Nevertheless, the utility of these cells for studies of cardiac hypertrophy signaling has recently been vividly demonstrated in a study of long-term stretch [59]. In brief, chronic cyclic stretch results in robust increases of myocyte growth, some but not all growth markers, and the expression of contractile proteins. In the present study, we have employed hESC-derived cardiomyocytes that can be maintained for periods of months in culture as a spontaneously beating functional syncytium. We have exposed these cultures to multiple known and hypothesized hypertrophic stimuli for prolonged periods, we have assessed structural changes and change of the expression of hypertrophy markers, such as alpha (α)-actinin and beta (β)-MHC, and we have determined that hypertrophy progression in multiple protocols is associated with both increased basal intracellular Ca^2+^ and increased DAG. Finally, we have addressed the question of whether basal (i.e., resting) myocyte Ca^2+^, as determined by the Na^+^ gradient, plays a role in the initiation of hypertrophy signaling in these cell cultures.

To reiterate the goal of this study, our central aim has been to characterize the progression of hypertrophy in human embryonic stem cell–derived cardiac myocytes in dependence on DAG and Na^+^ gradient changes, developing a case that positive feedback coupling between these mechanisms plays an important role in the initiation of hypertrophy programs. To this end, we have demonstrated that cardiac glycosides, which with strong certainty increase the resting cytoplasmic free Ca^2+^ by decreasing the Na^+^ gradient of myocytes [64], activate robust hypertrophy responses in hESC-derived myocytes and do not protect against hypertrophy caused by other agents at any concentration in this model. DAG, whether enhanced by classical cell signaling mechanisms or by inhibition of DAG lipase activity, also promotes similar myocyte growth responses. It therefore seems reasonable to suggest that the classical hormones that initiate hypertrophy programs rely on DAG-Ca^2+^ feedback mechanisms.

## Material and methods

### Cell culture and differentiation

Female embryonic stem cells (H9) cells are maintained in mTeSR1 (Stemcell Technologies, Cambridge, MA) on growth factor–reduced Matrigel-coated dishes. When H9 cells reach 85% confluency, cells are split 1:10 using EDTA solution (Versene) and maintained in mTeSR for 3 days until they reach ~ 85–90% confluency. Differentiation into human stem cell–derived cardiac myocytes is performed as described [6]. In brief, mTeSR is replaced with CDM3 consisting of RPMI 1640, 500 μg/mL recombinant human serum albumin (Oryzogen, Hubei, China), and 213 μg/mL L-ascorbic acid 2-phosphate (Wako, Richmond, VA). The medium is changed every other day (48 h). For days 0 to 2, CDM3 is supplemented with 6 μM CHIR99021 (Selleck Chemicals, Houston, TX); from day 2 to 4, the medium is changed to CDM3 supplemented with 2 μM Wnt-C59 (Selleck Chemicals, Houston, TX), and from day 4 to day 10, the CDM3 medium is changed every other day. Contracting cells are evident from day 7 on. From day 10 to 20, the medium is switched to RPMI 1640 without glucose supplemented with 2% B27 supplement; the medium is changed every other day. From day 21 on, cells are maintained in RPMI 1640 supplemented with 2% B27 supplement.

For chronic treatments, cells were seeded on growth factor–reduced Matrigel-coated glass cover slips or growth factor–reduced Matrigel-coated cell culture dishes and hypertrophic stimuli were added for a duration of 5 days with daily medium exchange. For life cell imaging, cells were grown on growth factor–reduced Matrigel-coated 35-mm glass bottom dishes (MatTek, Ashland, MA) for at least 1 week prior to experiments.

Cells were used between 2 and 4 months post differentiation, and all experiments were performed on cells originating from multiple differentiations.

### Preparation of free fatty acids

Palmitoleic acid (Nu-Check Prep, Waterville MN), palmitic acid, and myristic acid were dissolved in warm dH_2_O at 2× concentration. Fatty acid–free bovine serum albumin (Alfa Aesar, Haverhill, MA) was prepared at 2× concentration and warmed to 37 °C. Under constant stirring, the warm free fatty acid (FFA) mixture was added dropwise to the BSA. Aliquots were stored in glass vials at − 80 °*C.* The medium containing the FFA triple mix was sterile filtered using a 0.2-μm sterile filter.

### Immunoblotting

For protein isolation, cells were washed 3 times with ice-cold PBS and homogenized in ice-cold RIPA buffer (in mM: 150 NaCl, 50 Tris–HCl [pH 8.0], 5 EDTA, 1 EGTA; Triton X-100 1% [vol/vol], deoxycholate 0.5% [wt/vol], SDS 0.1% [wt/vol], and protease inhibitor cocktail from Roche (Basel, Switzerland)) and lysed for 1 h at 4 °C. Lysates were cleared at 20,000×*g* for 15 min and subjected to SDS-PAGE and subsequent immunoblotting. α-Actinin, actin, and GAPDH anti-sera were used at 1:1000, and β-MHC antiserum was used at 1:5000.

### Immunofluorescence

Immunofluorescence studies were performed as described elsewhere [13]. In brief, cells were fixed in 4% paraformaldehyde (Electron Microscopy Sciences, Hatfield, PA) in 1× PBS for 10 min, permeabilized in 0.1% Triton X-100 in PBS for 3 min, and blocked with 1.5% BSA and 5% donkey serum (Jackson Laboratories, Bar Harbor, ME) in 1× PBS for 1 h. Fixed monolayers were incubated with primary antibodies in 1.5% BSA and 5% donkey serum overnight (α-actinin 1:1000, β-MHC 1:500) at 4 °C. Then, after three times washing in 1× PBS, cells were incubated with the appropriate secondary antibodies (Jackson Laboratories, Bar Harbor, ME) for 1 h at room temperature. Cells were washed three times for 10 min in 1× PBS and mounted on glass slides using VECTASHIELD (Vector Laboratories, Burlingame, CA) containing DAPI. Images were obtained using a Zeiss Observer Z1 microscope equipped with an AxioCam MRm camera and a ×20 DIC objective (software: AxioVision (Release 4.8.2)). Per cover slip, a minimum of 10 pictures were taken and single-cell fluorescence intensities of single cells were analyzed using ImageJ. An average of 10 cells/picture were randomly picked and analyzed resulting in a minimum of 100 observations/experiment. Experiments were performed at least 3× independently. The final representation as fold changes over control was chosen to account for differences in absolute values of arbitrary fluorescence units (AFUs).

### Cell area measurements

Wheat germ agglutinin (WGA, Life Technologies, Carlsbad, CA) staining was performed according to the manufacturer’s instructions. In brief, cells were incubated for 10 min with 1 μg/mL WGA in HBSS at 37 °C. Subsequently, cells were fixed in 4% paraformaldehyde (Electron Microscopy Sciences, Hatfield, PA) in 1× PBS for 10 min and mounted on glass slides using VECTASHIELD containing DAPI. Images were obtained using a Zeiss Observer Z1 microscope equipped with an AxioCam MRm camera and a ×20 DIC objective (software: AxioVision (Release 4.8.2)). Per condition, a minimum of 10 pictures were taken. Analysis of cell area was carried out using ImageJ in a blinded fashion and with image regions selected in a random fashion. Pictures were thresholded, and area was measured and divided by the number of cells to obtain the average cell size.

### Calcium imaging

Cells were loaded with 5 μM Fluo-4 (Invitrogen, Carlsbad, CA) at 37 °C for 25 min. Subsequently, cells were washed 3× and placed in a buffer containing the following (in mM): 130 NaCl, 5 KCl, 10 HEPES, 1.6 MgCl_2_, 2 CaCl_2_, and 15 glucose, pH 7.4. Fifty micromolar of lidocaine were added to stop cell contractions. Cells were mounted on a Nikon Eclipse TE2000-S microscope equipped with a Photometrics CoolSNAP ES2 camera and a ×10 DIC objective, and the baseline Ca^2+^ was measured for 10 min at RT. Subsequently, cells were treated and placed at 37 °C for 15 min. Cells were mounted on the microscope again, and the same cell cluster was imaged for another 30 min. For maximal Ca^2+^ increases, 25 μM ferutinin was added. After background subtraction, data were normalized to the maximal signal and expressed as percentage of maximal arbitrary fluorescence units (AFUs). A minimum of 3 experiments was performed per condition.

### Patch clamp

Myocytes were patch clamped, and Na^+^/K^+^ pump currents were monitored as described previously [42, 43].

### DAG measurements

Cells were transduced with a DAG sensing protein (BacMam; Montana Molecular, Bozeman, MT) and grown for 7 days before being employed in experiments. Cells were washed and stimulated with a buffer containing the following (in mM): 130 NaCl, 5 KCl, 10 HEPES, 1.6 MgCl_2_, 2 CaCl_2_, and 15 glucose, pH 7.4. Cells were mounted on a Nikon Eclipse TE2000-S microscope equipped with a Photometrics CoolSNAP ES2 camera and a ×10 DIC objective, and the baseline DAG signal was measured for 10 min at RT. Subsequently, cells were treated and placed at 37 °C for 15 min. Cells were mounted on the microscope again, and the same cell cluster was imaged for another 30 min. For maximal DAG signals, 1 μM phorbol myristate acetate (PMA) was added. After background subtraction (baseline measurement), data were normalized to the maximal signal and expressed as percentage of maximal arbitrary fluorescence units (AFUs). A minimum of 3 experiments were performed per condition.

### Reagents and media

Cell culture reagents and media were obtained from Gibco/Life Technologies (Carlsbad, CA) unless otherwise stated. All other reagents were obtained from Millipore Sigma (Burlington, MA) in the highest available quality unless otherwise stated.

### Antibodies

Anti-actin antibody, clone C4 (Millipore Sigma MAB150), α-actinin (sarcomeric) antibody (Millipore Sigma A7732), anti-myosin (Skeletal, Slow) (Millipore Sigma M8421), and GAPDH antibody (G-9) (sc-365062; Santa Cruz, Dallas, TX).

### Statistics

Unless stated otherwise, error bars represent standard deviations. Significance was assessed by Student’s *t* test or, in occasional cases of inappropriate variance differences, by the Mann–Whitney rank-sum test.

## Results

### hESC-derived cardiomyocytes develop hypertrophy in response to AT-2 and ET-1

**Fig. 2:**
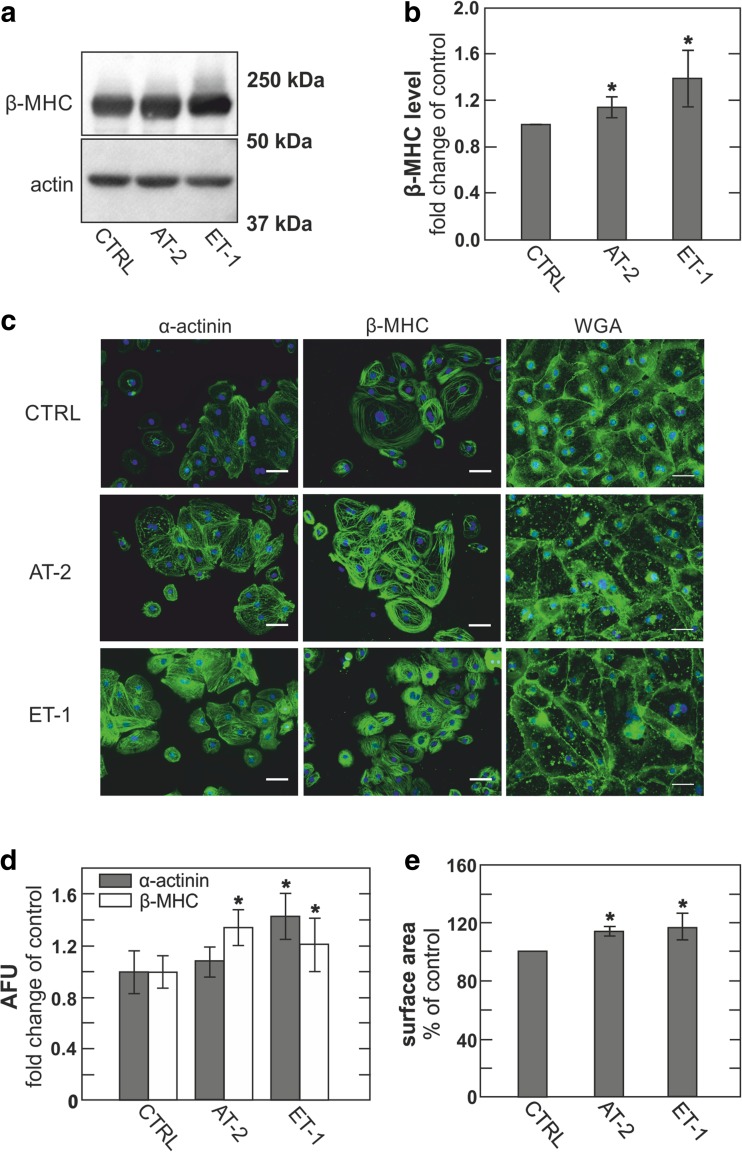
hESC-derived cardiomyocytes develop hypertrophy upon treatment with angiotensin-2 (AT-2) and endothelin-1 (ET-1). **a** Immunoblot of hESC-derived cardiomyocytes treated with 1 μM AT-2 and 25 pM ET-1 for 5 days. **b** Densitometric quantification of immunoblots normalized to actin and expressed as fold change compared to control, *n* = 3. **c** Representative micrographs of immunofluorescence and WGA staining of hESC-derived myocytes treated with vehicle, AT-II, or ET-1, against α-actinin, β-MHC. Scale bar 50 μm. **d** Quantification of immunofluorescence staining for α-actinin (gray) and β-MHC (white) expressed as fold change compared to control, *n* = 3. **e** Relative dimensions of hESC-derived cardiomyocytes treated with vehicle (CTRL), AT-2, or ET-1, expressed as fold change of control

As shown in Fig. 2a, b, immunoblots revealed clear increases in β-MHC expression levels in cultures treated with 1 μM AT-2 or 25 pM ET-1 versus control cultures treated with vehicle, i.e., 0.1% distilled water (dH_2_O), for 5 days. After fixation and immunofluorescent staining, analysis of the identically treated cultures revealed substantial increases in the expression of β-MHC in response to both agents (Fig. 2c, d). Treatment with ET-1, but not AT-2, resulted in marked increases in α-actinin expression (Fig. 2c, d). Further, we subjected hESC-derived cardiomyocytes to wheat germ agglutinin (WGA) staining and analyzed average dimensions of AT-2- and ET-1-treated cells compared to vehicle-treated control cells (Fig. 2c, e). Cell dimensions were robustly increased by both treatments in comparison to control cells.

### hESC-derived cardiomyocyte develop hypertrophy in response to exposure to a mixture of palmitic, myristic, and palmitoleic acid

**Fig. 3:**
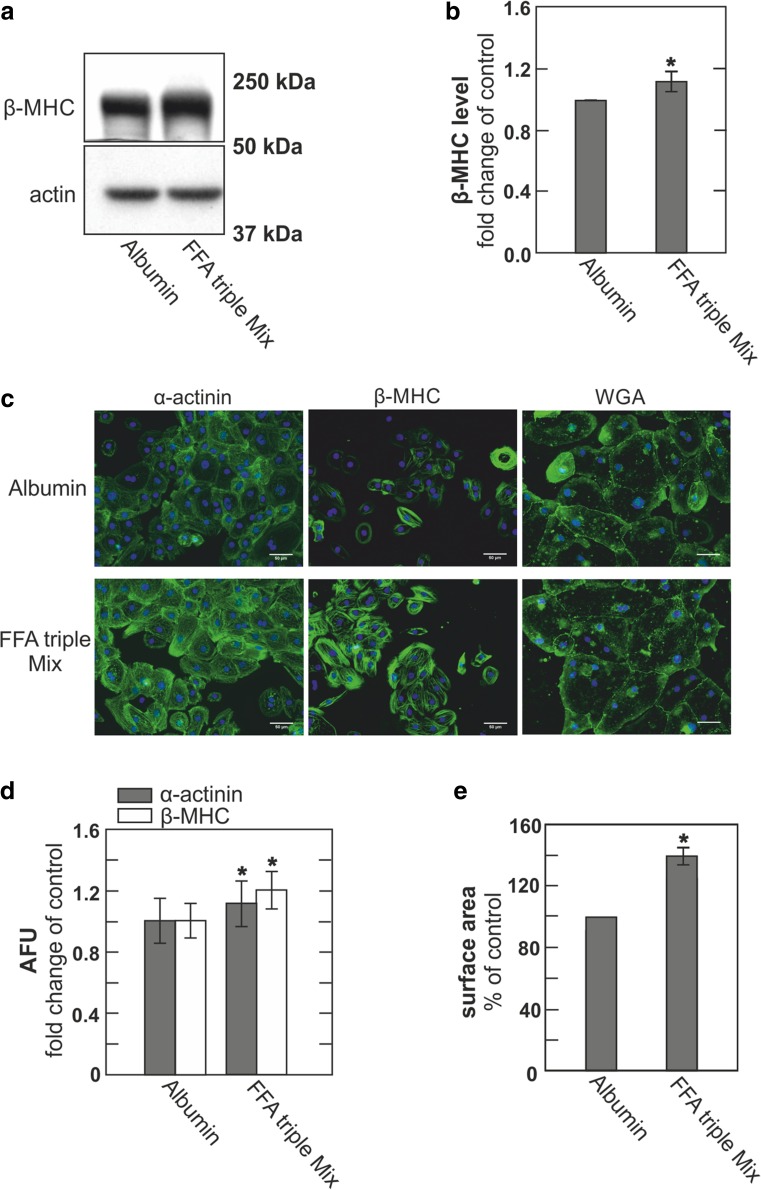
hESC-derived cardiomyocytes develop hypertrophy upon treatment with a triple FFA mix determined to cause cardiac myocyte growth in pythons and mice. **a** Immunoblot of hESC-derived cardiomyocytes treated with albumin or FFA triple mix for 5 days. **b** Densitometric quantification of immunoblots normalized to actin and expressed as fold change compared to control, *n* = 3. **c** Representative micrographs of immunofluorescent and WGA staining of hESC-derived cardiomyocytes treated with albumin or FFA triple mix. Scale bar 50 μm. **d** Quantification of immunofluorescence staining for α-actinin (gray) and β-MHC (white) expressed as fold change compared to control. **e** Relative dimensions of hESC-derived cardiomyocytes treated with albumin or FFA mix, expressed as fold change of control

Among the diverse stimuli well documented to induce cardiac hypertrophy, free fatty acids (FFAs) [65] are of special interest because they activate cardiac growth that is characterized as “beneficial” and are not well known to activate any of the common hypertrophy pathways. Next, therefore, we treated hESC-derived cardiomyocytes with a mixture of three FFAs that was identified to promote “beneficial” hypertrophy in hearts of the Burmese python, as well as in mice [65]. This mixture of palmitic, myristic, and palmitoleic acids, applied as equimolar complexes with bovine serum albumin (BSA) (0.2 mM in a 1:1:1:1 ratio), resulted in robust up-regulation of α-actinin and β-MHC as verified by both immunofluorescence and immunoblotting (Fig. 3a–d). Cell dimensions were also clearly increased in comparison to albumin-treated control cells, as determined in WGA-treated cells (Fig. 3c, e), and we describe subsequently that the effective FFA treatments increase both cytoplasmic Ca^2+^ and DAG levels.

### Inhibition of DAG lipase activity by DO34 causes hypertrophy in hESC-derived cardiomyocytes

**Fig. 4:**
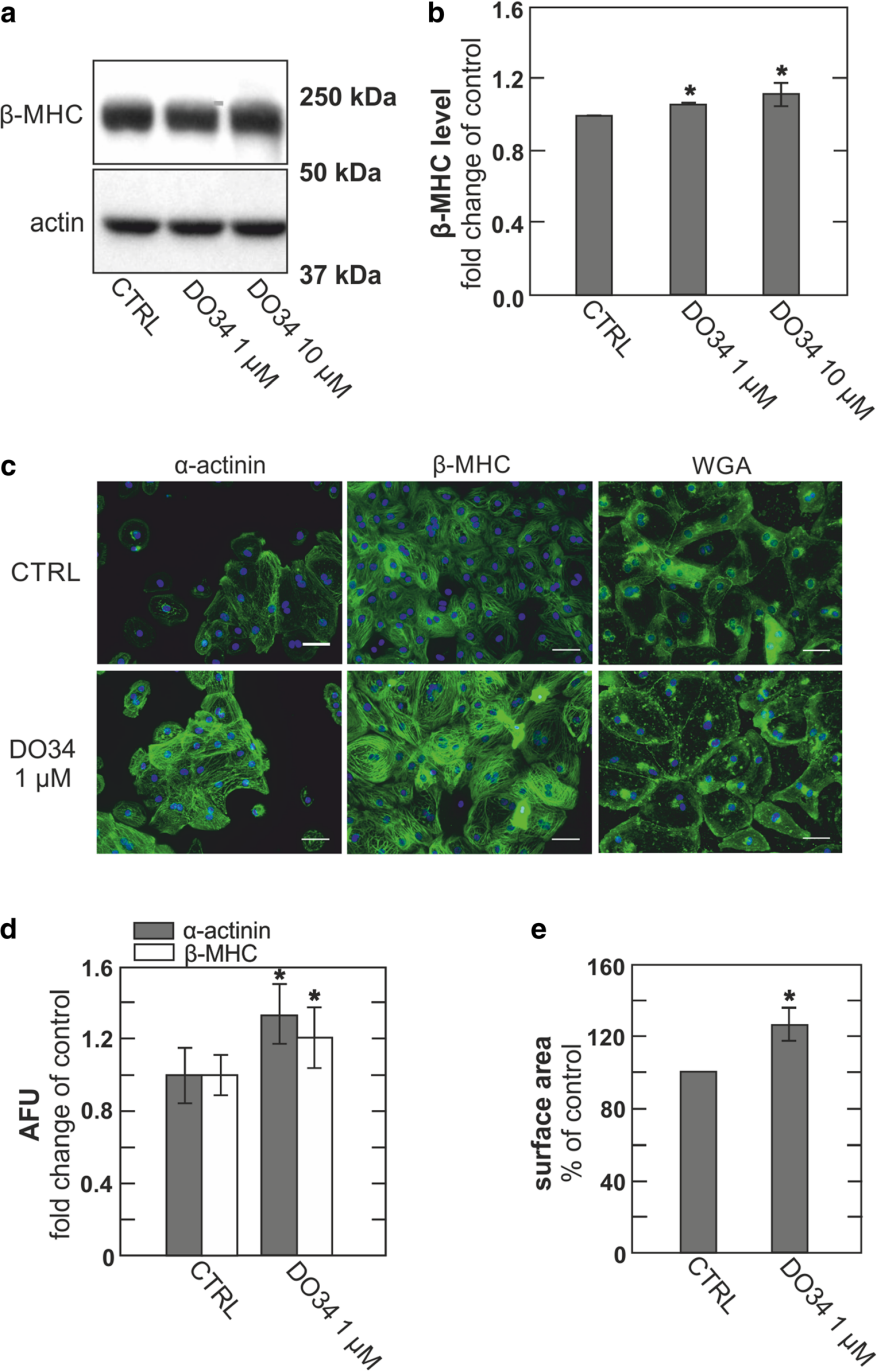
hESC-derived cardiomyocytes develop hypertrophy upon treatment with the DAG lipase inhibitor, DO34. **a** Immunoblot of hESC-derived cardiomyocytes treated with vehicle (CTRL) and different amounts of DO34 for 5 days. **b** Densitometric quantification of immunoblots normalized to actin and expressed as fold change compared to control, *n* = 3. **c** Representative micrographs of immunofluorescent and WGA staining of hESC-derived cardiomyocytes treated with vehicle (CTRL) or 1 μM DO34. Scale bar 50 μM. **d** Quantification of immunofluorescence staining for α-actinin (gray) and β-MHC (white) expressed as fold change compared to control. **e** Relative dimensions of hESC-derived cardiomyocytes treated with vehicle (CTRL) or 1 μM DO34 expressed as fold change of control

It is reasonably established that DAG signaling in cardiac myocytes is terminated by roughly equal activities of DAG lipases, cleaving DAG to monoacylglycerol and fatty acid, and DAG kinases that phosphorylate DAG to phosphatidate [10, 27]. A recently developed DAG lipase inhibitor, DO34, has profound effects on neuronal function that reflect with reasonable certainty changes of DAG metabolism [14]. Therefore, we tested whether increases in cellular DAG content through inhibition of DAG lipases, which mediate the hydrolysis of DAG, would result in hypertrophic responses in hESC-derived myocytes. To do so, we treated cells with 1 and 10 μM DO34 (Fig. 4a, b). Immunofluorescence analysis of cells treated with 1 μM DO34 confirmed the immunoblot data, indicating substantial hypertrophic responses. Both β-MHC and α-actinin staining were clearly increased (Fig. 4c, d), and 1 μM DO34 was further determined to significantly increase cell dimensions as analyzed after WGA staining (Fig. 4c, e).

### Low concentrations of ouabain (1 to 3 nM) have no cardioprotective effect in hESC-derived cardiomyocytes, while moderate ouabain concentrations (3 to 30 nM) cause hypertrophy

**Fig. 5:**
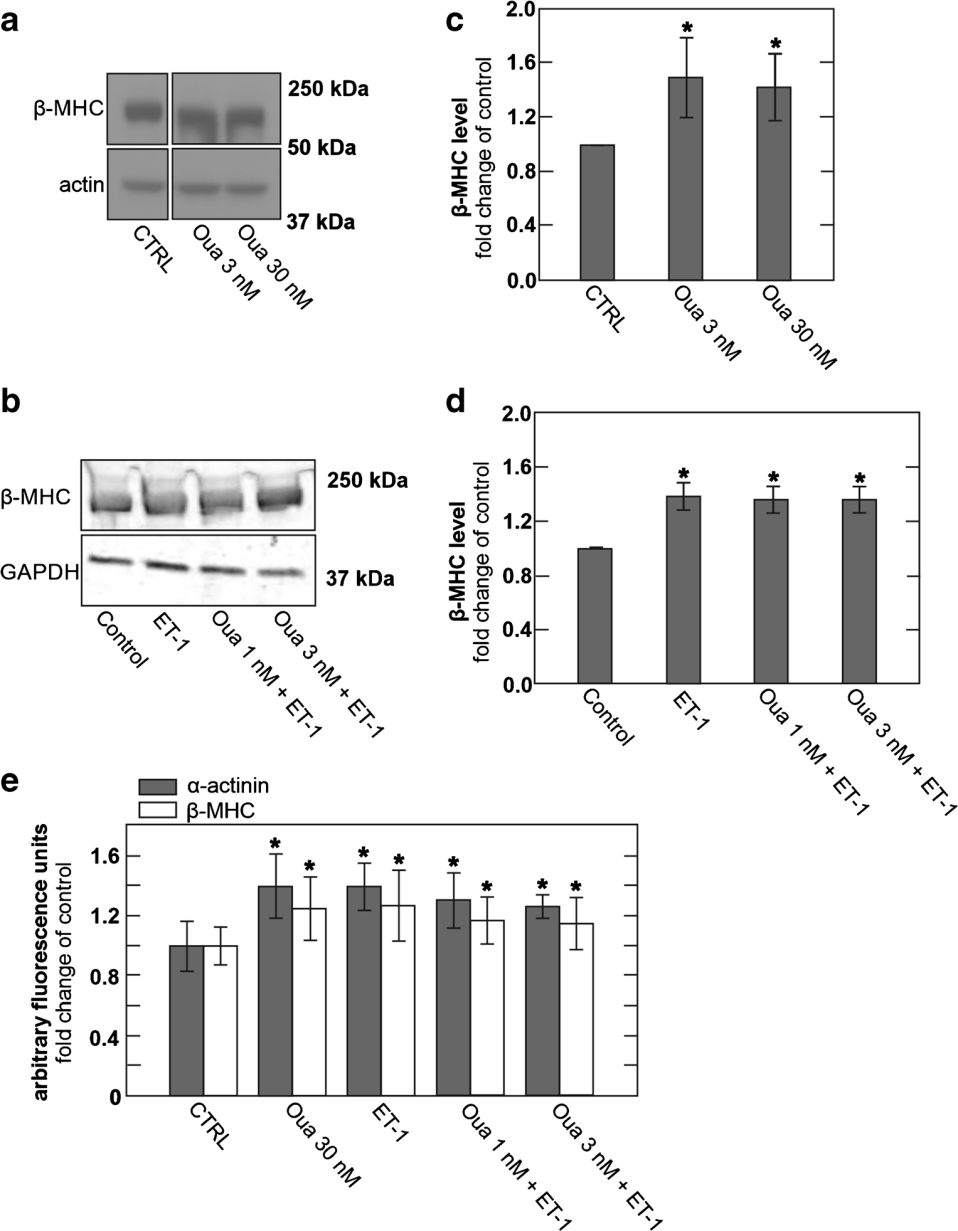
hESC-derived cardiomyocytes develop hypertrophy upon treatment with the heart glycoside ouabain. Low concentrations of ouabain do not block the hypertrophic response to ET-1. **a** Immunoblot of hESC-derived cardiomyocytes treated with vehicle (CTRL) or different concentrations of ouabain. **b** hESC-derived cardiomyocytes were treated with vehicle, ET-1, and 1 or 3 nM ouabain combined with ET-1 after a 12 h pre-incubation with low concentrations of ouabain alone. **c** Densitometric quantification of immunoblots of cells treated with 3 and 30 nM ouabain, respectively, normalized to actin and expressed as fold change compared to control, *n* = 3. **d** Densitometric quantification of immunoblots of cells treated with low concentrations of ouabain and ET-1 normalized to actin and expressed as fold change compared to control, *n* = 3. **e** Quantification of immunofluorescence staining for α-actinin (gray) and β-MHC (white) expressed as fold change compared to control, *n* = 3

A number of reports suggest that ouabain is a cardioprotective agent when employed in low nanomolar concentrations that putatively stimulate Na^+^/K^+^ pump activity [47, 62]. These results have led to a discussion that beneficial effects of heart glycosides, at low concentrations typically achieved in patients, might in fact be related to stimulation, rather than inhibition, of Na^+^/K^+^ pump activity [11, 21]. Therefore, we treated hESC-derived myocyte cultures with 1 and 3 nM ouabain, respectively, for 12 h, followed by application of hypertrophy inducing concentrations of 25 pM ET-1 for 4 days and determined whether the low ouabain concentrations might inhibit the development of hypertrophy as compared to control and endothelin-treated cultures. In contrast to this expectation, the up-regulation of hypertrophic markers by ET-1 was unaffected by 1 and 3 nM ouabain (Fig. 5b–e). Treatment of cultures with 3- and 30-nM concentrations of ouabain in the absence of ET-1 resulted in robust up-regulation of β-MHC expression (Fig. 5a, c), as well as pronounced increases in α-actinin and β-MHC staining determined by immunofluorescence (Fig. 5e).

**Fig. 6:**
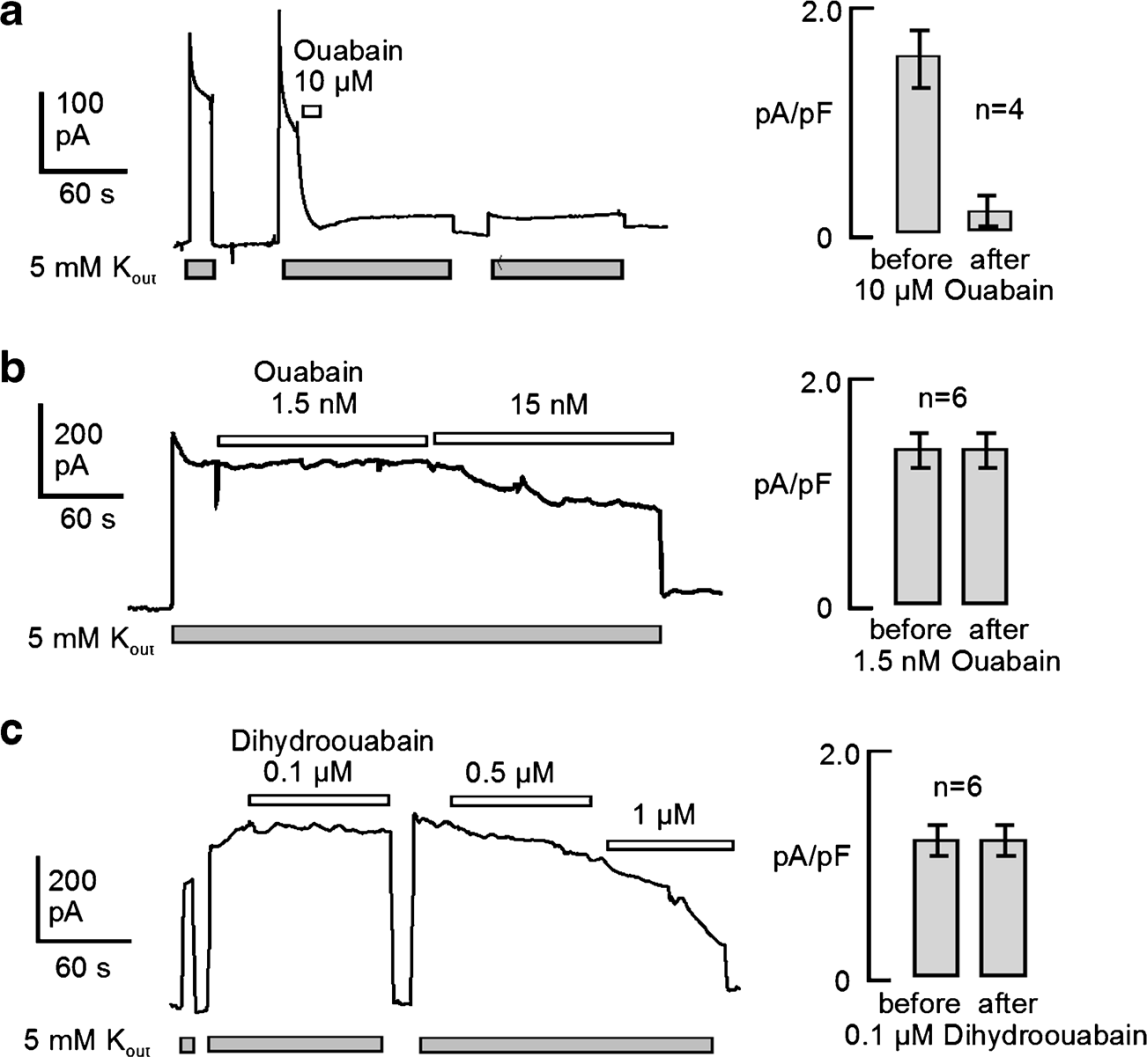
Activation of outward Na^+^/K^+^ pump current by application of extracellular K^+^ (5 mM), as indicated. Effects of ouabain on currents are quantified in the right panels. **a** Under standard experimental conditions for Na^+^/K^+^ pump currents, extracellular K^+^ was applied and removed three times. During the second application of K^+^, 10 μM ouabain was applied and the current was suppressed by 90% within ~ 5 s. The inhibitory effect persisted for more than 5 min after removing ouabain. On average, inhibition by 10 μM ouabain amounted to 90% of the steady state pump current. **b** Effects of progressive application of 1.5 and 15 nM ouabain. Concentrations of less than 2 nM did not cause detectable stimulation of Na^+^/K^+^ pump currents, and higher concentrations were exclusively inhibitory. In 6 experiments, application of 1.5 nM ouabain caused no discernible effect on pump currents. **c** Effects of progressive application of 0.1, 0.5, and 1 μM dihydroouabain. Concentrations of less than 0.5 μM did not cause detectable stimulation of Na^+^/K^+^ pump currents, and higher concentrations were exclusively inhibitory

Given this outcome, we next attempted to verify that low-dose ouabain could stimulate Na^+^/K^+^ pump activity in hESC myocytes. In these recordings, we defined the magnitudes of Na^+^/K^+^ pump currents in hESC-derived myocytes, removed from dishes, and their responses to different concentrations of ouabain. The recording conditions were identical to those employed in recent Na^+^/K^+^ pump studies of murine myocytes [42, 43] using 25 mM cytoplasmic Na^+^ and 120 mM extracellular Na^+^. As illustrated in Fig. 6a, application of 10 μM ouabain rapidly inhibited Na^+^/K^+^ pump currents activated by 5 mM K^+^ by about 90% and the inhibitory effect did not reverse after several minutes of superfusion with ouabain-free solution. To determine whether pump activity could be stimulated by low ouabain concentrations, we first examined effects of ouabain concentrations from 0.5 to 15 nM. We were unable to detect any effect of ouabain at concentrations less than 5 nM, and we observed only inhibitory effects at higher concentrations. This is illustrated in the series of experiments shown in Fig. 6b in which first 1.5 nM and then 15 nM were applied. As quantified in the bar graphs, 1.5 nM was without effect, and 15 nM was inhibitory. Since the dissociation of ouabain from these cells clearly requires many minutes, we next examined whether the lower-affinity heart glycoside, dihydroouabain (e.g., [44]), might exert a stimulatory effect on pump currents in these cells. As illustrated in Fig. 6c, we employed increasing concentrations of dihydroouabain and observed only inhibitory effects. As shown in the bar graphs, 0.1 μM dihydroouabain was without effect, whereas inhibitory effects were observed at 0.5 μM and 1 μM.

### Acute treatment with diverse hypertrophic stimuli causes increases in both basal intracellular Ca^2+^ and DAG

**Fig. 7:**
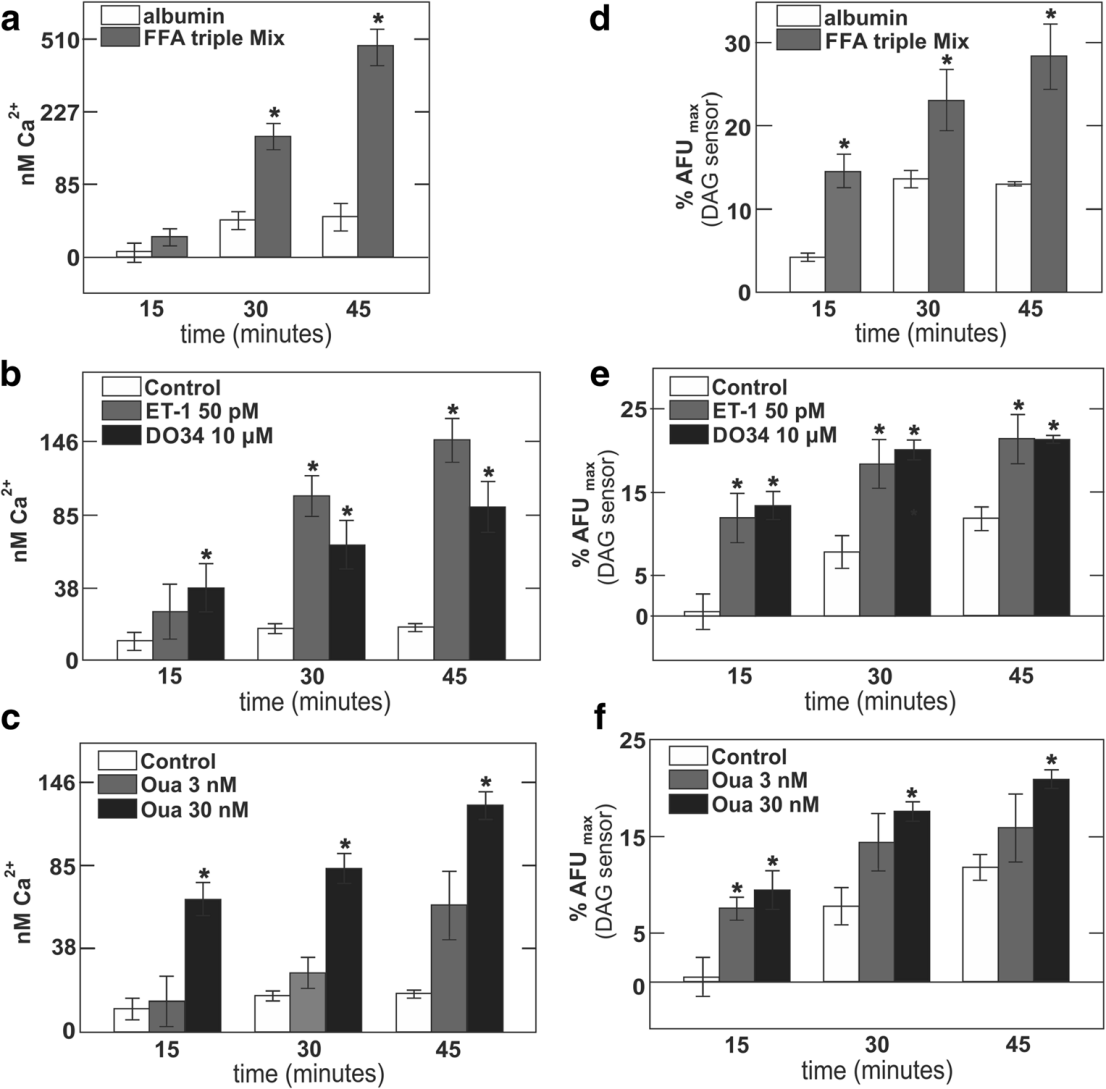
Acute treatment of hESC-derived cardiomyocytes with hypertrophic agents causes significant increases of both intracellular Ca^2+^ and DAG. **a** Cytoplasmic Ca^2+^ increases over 45 min in albumin (white) and FFA triple mix (gray) treated cells, *n* = 3. **b** Intracellular Ca^2+^ increases over 45 min in vehicle (white), ET-1-treated (gray), and DO34-treated (black) cells, *n* = 3. **c** Intracellular Ca^2+^ increases over 45 min in vehicle (white), 3 nM ouabain (gray), and 30 nM ouabain (black) treated cells, *n* = 3. **d** Increases in intracellular DAG levels over 45 min in albumin (white) and FFA triple mix (gray) treated cells, *n* = 3. **e** Increases in intracellular DAG levels over 45 min in vehicle (white), ET-1-treated (gray), and DO34-treated (black) cells, *n* = 3. **f** Increases in intracellular DAG levels over 45 min in vehicle (white), 3 nM ouabain-treated (gray) and 30 nM ouabain-treated (black) cells, *n* = 3

To test the working hypothesis that Ca^2+^ and DAG support hypertrophic growth in a positive feedback manner, we next analyzed the acute effects of several hypertrophic stimuli on the steady-state cytoplasmic Ca^2+^ and DAG levels in hESC-derived cardiomyocytes. To measure intracellular Ca^2+^, cells were loaded with the non-ratiometric Ca^2+^ dye, Fluo-4 [22], and to measure cytosolic DAG, cells were transduced with a FRET-based DAG sensing protein [73]. Ca^2+^ and DAG measurements were carried out at multiple time points over a period of 45 min after addition of hypertrophic stimuli or vehicle. After 45 min, the nonselective ionophore, ferutinin (25 μM) [82], was applied to determine the maximal Ca^2+^ response of Fluo-4, while 1 μM PMA was employed to determine the maximal DAG (C1 domain) response. The calibration of free Ca^2+^ concentrations, given in Fig. 7, is based on a *K*_d_ of Fluo-4 for Ca^2+^ of 340 nM [22] and the assumption that maximal fluorescence was achieved during ferutinin treatment. As shown in Fig. 7a, the FFA mix over 45 min resulted in an increase in cellular basal Ca^2+^ from less than 40 nM to about 500 nM, whereas Ca^2+^ in vehicle (albumin)-treated cells rose to just 50 nM. As shown in Fig. 7d, DAG levels were rapidly increased by 3-fold over control levels and to 28% of maximal FRET responses over 45 min, while DAG signals in control cells rose to 12% of maximal FRET responses. For ET-1 (50 pM) and the DAG lipase inhibitor, DO34 (10 μM), shown in Fig. 7b, e, basal Ca^2+^ rose over 45 min to 90 and 145 nM, respectively. During the same time frame, control basal Ca^2+^ levels remained at about 20 nM. DAG levels in both treatments rose to 21% of maximal FRET responses, whereas control DAG levels rose to 13% of maximal FRET responses.

Results for treatment of cell cultures with a low ouabain concentration (3 nM) and a moderate ouabain concentration (30 nM) are shown in Fig. 7c, f. From our electrophysiological studies, we project that these ouabain concentrations will result in distinct inhibition of Na^+^/K^+^ pumps in these cells (Fig. 6). The lower concentration (3 nM) led to a basal Ca^2+^ estimated to be 57 nM, compared to 22 nM for control cells, and 120 nM for cells treated with the higher ouabain concentration (30 nM). DAG levels rose to 15 and 21% for low and high ouabain concentrations, respectively, compared to 11% of maximal FRET for control cells. We mention in closing the “Results” section that increases in DAG mass have been described previously in exercise-induced hypertrophy models but not in pressure overload [15].

## Discussion

This study documents further the utility and impressive advantages of hESC-derived cardiomyocytes for long-term studies of cell signaling mechanisms in cardiac myocytes. Obviously, the ability to maintain spontaneously beating syncytial human myocytes for periods of months in culture, in the absence of fibroblasts, is highly promising for a wide range of studies of long-term myocyte signaling programs, such as those originally envisioned by the Alliance for Cell Signaling [23]. Our results support a working hypothesis that positive feedback coupling between Ca^2+^ and DAG signaling plays an important role in the initiation of cardiac hypertrophy programs. Specifically, we have demonstrated that basal Ca^2+^ and DAG increase to similar extents in remarkably diverse treatments that promote hypertrophy. Interventions expected to increase primarily Ca^2+^ increase DAG substantially and vice versa. This outcome has strong implications for an understanding of cardiac excitation–contraction coupling as well as the initiation of cardiac hypertrophy programs.

Largely as expected, hESC-derived cardiomyocytes show substantial hypertrophy responses over several days during treatment with AT-2 and ET-1 (Fig. 2). The magnitudes of changes of hypertrophy markers are comparable to responses described in other hypertrophy studies of hESC-derived myocytes [19, 59]. However, it is noteworthy that cyclic stretch caused hypertrophy without a clear change of β-MHC [59]. While changes of β-MHC and other hypertrophy markers are often larger in conventional myocyte hypertrophy models (e.g., [8, 83]), that clearly is not always the case (e.g., [61, 67]). Both the time required (3 to 5 days) and magnitudes of responses are reasonable with respect to previous work. Although equivalent responses in intact mammals besides rodents can require one to multiple weeks (e.g., in dogs, [24]), cultured muscles from rabbits, for example, have been shown to develop significant hypertrophy within 24 to 48 h of treatments with AT-2 or ET-1 [5]. Notably, an essential role for DAG-dependent activation of PKCs and subsequently ERK1/2 activation was demonstrated for hypertrophy responses in this latter study, whereas a role for IP_3_ receptors was negated. That AT-2 and ET-1 can increase Ca^2+^ transients and promote activation of specific PKCs in cardiac myocytes, notably PKCε [33], is well documented and accepted [68, 84]. We are however not aware that previous work documented increases of DAG, per se, in the membranes of cardiac myocytes with these agonists. We note in this connection that the DAG sensor employed in this study responds to binding DAG with an increase in FRET [73], and that our measurements therefore do not rely on a translocation of the sensor to the membrane as in other C1 domain–based DAG assays [1]. Furthermore, it has remained an important question as to whether diastolic, basal Ca^2+^ in myocytes is increased by these reagents. Our results show unambiguously that both AT-2 and ET-1 increase basal Ca^2+^, determined after fully blocking spontaneous action potential firing (Fig. 7).

These results indicate that growth-inducing hormones can be acting by increasing Ca^2+^ influx through channels that are open during diastole (e.g., TRPC channels) and/or may be acting by increasing the resting Na^+^ concentration of myocytes with concomitant rises in cytoplasmic Ca^2+^ by Na^+^/Ca^2+^ exchange. Consistent with the idea that a rise in resting Na^+^ can promote hypertrophy responses in these cells, ouabain induces robust hypertrophy responses in these myocytes at concentrations that inhibit Na^+^/K^+^ pump activity (Fig. 6). These results bring Na^+^ homeostasis to the forefront as an important factor in the initiation of pathological cardiac hypertrophy. In fact, a major role of the Na^+^/H^+^ exchanger, NHE1, in contributing Na^+^ load that promotes the initiation of cardiac hypertrophy, as well as its progression to cardiac failure, has long been indicated [18, 25, 34, 48]. It will be of considerable interest to determine why stimulatory effects of low ouabain concentrations were not observed in these human cardiomyocytes, specifically whether this failure is related to the Na^+^/K^+^ pump isoforms expressed in these cells and/or whether the cellular conditions of our experiments and the differentiation state of myocytes might play critical roles.

To evaluate the potential triggering role of DAG per se, versus IP_3_, in initiating hypertrophy responses in these myocytes, we employed a new inhibitor of DAG lipases that profoundly affects brain function [7, 56]. The results show clearly that lipase inhibition causes hypertrophy responses that are comparable to those induced by AT-2 and ET-1 (Fig. 4). They show further that both Ca^2+^ and DAG signaling becomes enhanced. These results support the idea that the termination of DAG signaling can be importantly influenced by DAG lipase activity in cardiac myocytes, as well as by the parallel function of DAG kinases that is already established to control the initiation of hypertrophy programs [55]. How a mix of saturated and unsaturated FFA (Fig. 3) might induce similar phenotypes as a DAG lipase inhibitor (Figs. 4 and 7) remains an open question. Inhibition of DAG lipase activity would be one possible mechanism of FFA action. However, the simplest mechanism would be enhanced de novo synthesis of DAG, i.e., from FFA and glycerol-3-phosphate, which has indeed been shown to modify DAG-dependent cell signaling mechanisms [9]. We point out in this connection that polyunsaturated FFAs on their own suppress cardiac hypertrophy, possibly by promoting the expression of the DAG kinase ξ isoform [30].

It is especially notable that the increases in Ca^2+^ occurring with DAG lipase inhibition are of similar magnitude to those that occur with ET-1, and that changes in DAG with DAG lipase inhibition are not notably greater than those occurring with hormones (Fig. 7). These outcomes suggest that the coupling between DAG and Ca^2+^ signaling mechanisms is quite strong over substantial signaling ranges. One factor that may contribute to coordinated Ca^2+^ and DAG signaling is that these hESC myocyte cultures are well coupled electrically and therefore behave in a substantially more coordinated fashion than traditional myocyte cultures used in hypertrophy studies. Another key factor may be that DAG acts primarily to increase cytoplasmic Na^+^ influx via TRP channels, rather than enhancing Ca^2+^ influx per se. In contrast to a direct Ca^2+^ influx mechanism, the accumulation of cytoplasmic Na^+^ may be expected to inhibit Ca^2+^ extrusion via Na^+^/Ca^2+^ exchange and thereby enhance the actions of all mechanisms that tend to increase myocyte Ca^2+^ signals.

In summary, the present study highlights the potential importance of self-amplifying signaling via Ca^2+^- and DAG-dependent mechanisms in the initiation of cardiac hypertrophy programs. Ouabain-dependent signaling mechanisms may well modify this coupling and play some role in the outcomes observed. Certainly, it will be important to delineate the role specifically of Ca-independent ouabain signaling mechanisms [40, 78]. In the first order, however, our results support that well-described cell signaling mechanisms support the proposed positive feedback, and it seems difficult to deny that this signaling axis will play a major role in initiating hypertrophy programs. Finally, we stress that the hESC-derived myocytes employed in this study are clearly well suited to develop genetic models to address all of the questions raised by this study. Our results therewith underscore the great experimental potential of hESC-derived myocytes and should promote their further development. The fact that low, “therapeutic” concentrations of heart glycosides induce robust hypertrophy phenotypes in these human myocytes underscores that Na^+^ homeostatic mechanisms are likely at play constitutively in the control of cardiac signaling and myocyte phenotype.
